# Retraction Note: Selinexor (KPT-330) has antitumor activity against anaplastic thyroid carcinoma in vitro and in vivo and enhances sensitivity to doxorubicin

**DOI:** 10.1038/s41598-025-31415-1

**Published:** 2025-12-15

**Authors:** Manoj Garg, Deepika Kanojia, Anand Mayakonda, Trivadi S. Ganesan, Bindhya Sadhanandhan, Sidhanth Suresh, Sneha S., Rohit P. Nagare, Jonathan W. Said, Ngan B. Doan, Ling-Wen Ding, Erkan Baloglu, Sharon Shacham, Michael Kauffman, H. Phillip Koeffler

**Affiliations:** 1 Cancer Science Institute (CSI) of Singapore, National University of Singapore, Singapore, Singapore; 2 Department of Medical Oncology and Clinical Research, Cancer Institute (WIA), Adyar, Chennai India; 3 Department of Pathology and Laboratory Medicine, David Geffen School of Medicine, Los Angeles, CA USA; 4 Karyopharm Therapeutics Inc, Newton, MA 02459 USA; 5 Division of Hematology/Oncology, Cedars-Sinai Medical Center, University of California Los Angeles, School of Medicine, Los Angeles, CA USA

Retraction of: *Scientific Reports* 10.1038/s41598-017-10325-x, published online 29 August 2017

The Editors have retracted this Article.

After publication of this Article, concerns were raised regarding some image irregularities and data concerns. Specifically:

- Figure 4D, Vehicle, tunel antibody in this Article and Figure 4D, Vehicle, tunel antibody in^1^ appear to be highly similar;
- Figure 4B appears to have signs of digital editing and there appear to be a discrepancy between the tumours in the image and the quantification of the tumour weights;
- Supplementary Figure 3B, DMSO (HTH83) and Supplementary Figure 3B, 125 nM (HTH83) appear to be highly similar and to present signs of digital editing;
- Supplementary Figure 3B, DMSO (CAL62) and Supplementary Figure 3B, 125 nM (CAL62) appear to be highly similar and to present signs of digital editing.

In addition, Figure 2C, Cal62 (0-63-125-250-500-1000) and Figure 2A, SW872 (0-125-250-500-1000-2000) in^1^ appear to be highly similar.

Upon request, the Authors were able to provide the raw data for Figure 2C but not for the other image concerns, and were not able to provide a satisfactory response to the concerns raised. The Editors no longer have confidence in the presented data.

Manoj Garg and Trivadi S Ganesan disagree with this retraction.

Deepika Kanojia, Anand Mayakonda, Bindhya Sadhanandhan, Sidhanth Suresh, Sneha S., Rohit P. Nagare, Jonathan W. Said, Ngan B. Doan, Ling-Wen Ding, Erkan Baloglu, Sharon Shacham, Michael Kauffman and H. Phillip Koeffler did not respond to correspondence from the Publisher about this retraction.
